# Identifying Imaging Markers for Predicting Cognitive Assessments Using Wasserstein Distances Based Matrix Regression

**DOI:** 10.3389/fnins.2019.00668

**Published:** 2019-07-10

**Authors:** Jiexi Yan, Cheng Deng, Lei Luo, Xiaoqian Wang, Xiaohui Yao, Li Shen, Heng Huang

**Affiliations:** ^1^School of Electronic Engineering, Xidian University, Xi'an, China; ^2^Electrical and Computer Engineering, University of Pittsburgh, Pittsburgh, PA, United States; ^3^Department of Biostatistics, Epidemiology and Informatics Perelman School of Medicine, University of Pennsylvania, Philadelphia, PA, United States

**Keywords:** Alzheimer's disease, cognitive assessment, Wasserstein distance, matrix regression, feature selection

## Abstract

Alzheimer's disease (AD) is a severe type of neurodegeneration which worsens human memory, thinking and cognition along a temporal continuum. How to identify the informative phenotypic neuroimaging markers and accurately predict cognitive assessment are crucial for early detection and diagnosis Alzheimer's disease. Regression models are widely used to predict the relationship between imaging biomarkers and cognitive assessment, and identify discriminative neuroimaging markers. Most existing methods use different matrix norms as the similarity measures of the empirical loss or regularization to improve the prediction performance, but ignore the inherent geometry of the cognitive data. To tackle this issue, in this paper we propose a novel robust matrix regression model with imposing Wasserstein distances on both loss function and regularization. It successfully integrate Wasserstein distance into the regression model, which can excavate the latent geometry of cognitive data. We introduce an efficient algorithm to solve the proposed new model with convergence analysis. Empirical results on cognitive data of the ADNI cohort demonstrate the great effectiveness of the proposed method for clinical cognitive predication.

## 1. Introduction

Alzheimer's disease (AD), the most common form of dementia, is a Central Nervous System (CNS) chronic neurodegenerative disorder with progressive impairment of learning, memory and other cognitive function. As an incurable disease which severely impacts human thinking and behavior, Alzheimer's disease is the 6th cause of death in the United States (Alzheimer's Association, 2018). Along with the rapid progress in high-throughput genotype and brain image techniques, neuroimaging has been developed to effectively predict the progression of AD or cognitive performance in plentiful research (Ewers et al., 2011; Wang et al., 2011b), which benefits for early diagnosis and explorition of brain function associated with AD (Petrella et al., 2003; Avramopoulos, 2009). The Alzheimer's Disease Neuroimaging Initiative (ADNI) (Mueller et al., 2005; Jack et al., 2008) provides neuroimaging and cognitive measurement of normal aging, mild cognitive impairment as well as AD samples, which provides a wealth of resources for the study of Alzheimer's diagnosis, treatment and prevention.

Until now, numerous studies (Eskildsen et al., 2013; Moradi et al., 2015) have utilized neuroimaging techniques to detect pathology associated with AD. Among them, structural magnetic resonance imaging (MRI) is the most extensively used imaging modality in AD related studies because of its completely non-invasive nature, high spatial resolution, and high availability. Thus, researchers have extracted plentiful MRI boimarkers in classifying AD patients in different disease over the past few years (Duchesne et al., 2008; Eskildsen et al., 2013; Guerrero et al., 2014). And these abundant MRI boimarkers have been used to many AD related studies, such as AD status prediction and MCI-to-AD conversion prediction. Despite of great efforts, we still cannot identify informative AD-specific biomarkers for the early diagnosis and prediction of disease progression. The reason for this is that the number of clinical status of AD is small, which makes it difficult to observe and understand the cognitive progression.

Consequently, many studies use clinical cognitive tests to measure cognitive assessment. Recently, several clinical tests have been presented to access individual's cognitive level, such as Trail making test (TRAILS) and and Rey Auditory Verbal Learning Test (RAVLT) (Schmidt, 1996). Through predicting the cognitive scores with MRI biomarks, we can explore the association between imaging biomarkers and AD and find informative AD-specific biomarkers. Therefore, a wide range of machine learning approaches have been proposed to predict the cognitive scores and uncover the pathology associated with AD (Wang et al., 2011a, 2016; Moradi et al., 2017).

In the current study of predicting cognitive scores with longitudinal phenotypic markers extracted from MRI data, regression method has been demonstrated as a effective way to excavate the correlation between cognitive measures. To modify the traditional regression model, recent methods proposed to integrate novel regularization term (such as sparse regularization and low-rank regularization) into the traditional regression model (Obozinski et al., 2010; Jie et al., 2015; Moradi et al., 2017). In fact, the intrinsic idea of the study mentioned above is utilizing different matrix norm or the combination of matrix norms as the similarity measures of the empirical loss or regularization to fit the prior assumption of neuroimaging markers. Though the effectiveness of specific matrix norm as regularization, these matrix norms simply meet the assumption rather than make full use of the inherent geometry of the data. Thus, it is easy to achieve a suboptimal solution for these models.

To tackle this problem, in this paper we consider Wasserstein distance as distance metric for regression model. Different from *L*_*p*_ distances (*p* ≥ 0) (Luo et al., 2017) or Kullback-Leibler (Csiszár and Shields, 2004) and other *f*-divergences (Ali and Silvey, 1966), Wasserstein distance is well-defined between any pair of probability distributions over a sample space equipped with a metric. Thus, it provides a meaningful notion of distance for distributions supported on non-overlapping low dimensional manifolds. For better performance of cognitive score predication, we propose to substitute Wasserstein distance for matrix norm.

Although successfully applied to image retrieval (Rubner et al., 2000), contour matching (Grauman and Darrell, 2004), cancer detection (Ozolek et al., 2014), super-resolution (Kolouri and Rohde, 2015), and many other problems, there is an intrinsic limitation of Wasserstein distances. In fact, Wasserstein distances are defined only between measures having the same mass, which makes it difficult to applied Wasserstein distance into cognitive score prediction. To overcome such a limitation, many existed study (Piccoli and Rossi, 2014, 2016; Kondratyev et al., 2016), have been proposed. However, these methods are all based on distributions or histogram features of data. As we know, in cognitive score prediction, we usually use the original features rather histogram features to learn the regression model parameters. Additionally, most of these methods use traditional matrix norm to characterize model parameters in Wasserstein distance loss minimization problem. This often leads to suboptimal results since matrix norm is usually sensitive to real noise.

To perfectly integrate Wassterstein distance into regression model for better performance of cognitive score prediction, in this paper we propose a novel efficient and robust Matrix Regression method to employ Joint Wasserstein distances minimization on both loss function and regularization (JWMR for short). Different from the existing methods, which need to extract histogram features of data in the preprocessing stage and then calculate Wasserstein distances based on them, our method considers histogram operator as an important component of objective function and uses it to constrain loss term and the estimated model parameters which are generated by original data features. This is the first time for exploiting Wasserstein distance as loss and regularization terms. As a result, our method is more reliable and applicable than traditional regression method using ℓ_*p*_-norm regularizer. We derive an efficient algorithm based on a relaxed formulation of optimal transport, which iterates through applications of alternating optimization. We provide the convergence analysis of our algorithm and describe a statistical bound for the proposed new model. We apply our method on cognitive data of the ADNI cohort and obtain promising results.

Our main contributions are three-fold: (1) The proposed robust matrix regression via joint Wasserstein distances minimization to circumvent the natural limitation of matrix norms in regression model; (2) The proposed model is suitable for revealing the relationship between cognitive measures and neuroimaging markers; (3) Because our method not only includes composition of *W*(·, ·), but also the computations of Wasserstein distances with regard to different terms, we derive an efficient algorithm to solve this problem with convergence analysis.

## 2. Study of Cognitive Score Prediction

### 2.1. Notations

We summarize the notations and definitions used in this paper. Matrices are written as boldface uppercase letters. ∥·∥_*F*_ and ∥·∥_*_ denote Frobenius norm and nuclear norm, respectively. 〈·, ·〉 is the inner product operation. **e** ∈ ℝ^*m*^ is a column vector of ones. **0** ∈ ℝ^*m*^ is a column vector of zeros. For vector **m** ∈ ℝ^*m*^, its *i*-th element is denoted by *m*_(*i*)_. For matrix **M** ∈ ℝ^*n* × *m*^, its *i*-th row, *j*-th column and (*i, j*)-th element are denoted by **m**^*i*^, **m**_*j*_, and *m*_*ij*_. The ℓ_2,1_-norm of **M** is defined as

(1) $$\|M{\|}_{2,1}=\sum_{i=1}^{n}\sqrt{\sum_{j=1}^{m}m_{ij}^{2}}=\sum_{i=1}^{n}\|m^{i}{\|}_{2},$$

where $∥{\text{m}}^{i}{∥}_{2}$ denotes the ℓ_2_-norm of the vector **m**^*i*^. We define the Kullback-Leibler (KL) divergence between two positive vectors by

(2) KL(x,y)=〈x,log(x/y)〉+〈y−x,e〉,

where / denotes the element-wise division.

### 2.2. Matrix Regression for Cognitive Score Prediction

In the association study of predicting cognitive scores from imaging markers, a wide range of work has employed regression models to uncover the relationship between neuroimaging data and cognitive test scores and predict cognitive score. Given the imaging feature matrix **A** ∈ ℝ^*m* × *l*^ and the cognitive score matrix **Y** ∈ ℝ^*l* × *n*^, a common paradigm for regression to predict cognitive score is to minimize the penalized empirical loss:

(3) $${\text{min}}_{Z}L\left(Y-A^{T}Z\right)+\lambda \Omega \left(Z\right),$$

where λ > 0 is the balance parameter, **Z** ∈ ℝ^*m* × *n*^ is the weight matrix, which is estimated from the imaging feature matrix **A** and the cognitive score matrix **Y** to capture the relevant features for predicting the cognitive scores, $L\left(\text{Y}-{\text{A}}^{T}\text{Z}\right)$ is the empirical loss on the training set, and Ω(**Z**) is the regularization term that encodes imaging feature relatedness. Different assumptions on the loss $L\left(\text{Y}-{\text{A}}^{T}\text{Z}\right)$ and variate **Z** lead to different models. The representative model include:

Least Squares Regression (LSR) (Lu et al., 2012):

(4) $${\text{min}}_{Z}\|Y-A^{T}Z{\|}_{F}^{2}+\lambda \|Z{\|}_{F}^{2},$$

Low Rank Representation (LRR) (Liu et al., 2010):

(5) $${\text{min}}_{Z}\|Y-A^{T}Z{\|}_{1}+\lambda \|Z{\|}_{*},$$

Feature Selection Based on ℓ_2,1_-norm (Nie et al., 2010):

(6) $${\text{min}}_{Z}\|Y-A^{T}Z{\|}_{2,1}+\lambda \|Z{\|}_{2,1}.$$

### 2.3. Feature Selection for Informative Imaging Marker Identification

Due to the progress and prosperity of brain imaging and high-throughput genotyping techniques, a large amount of brain imaging data is available and a great quantity of imaging markers is alternative to predict cognitive score. However, not all of them are related to the pathological changes specific to AD, namely some imaging markers are redundancy for the prediction task. A forthright method to tackle this problem is to perform feature selection, which aims to choose a subset of informative features for improving prediction.

Feature selection has been demonstrated as a efficient way to reflect the correlation between cognitive measures after removing the non-distinctive neuroimaging markers. Regression techniques with specific regularization can also used to identify discriminative imaging markers. For instance, sparse regression models have been extensively utilized to select discriminative voxels for AD study in previous works (Guerrero et al., 2014; Liu et al., 2014; Xu et al., 2017). Many sparse-inducing norm have been iterated into the spare regression model: ℓ_1_ shrinkage methods such as LASSO can identify informative longitudinal phenotypic markers in the brain that are related to pathological changes of AD (Liu et al., 2014); group LASSO with a ℓ_2,1_-norm can select the most informative imaging markers related to all participants including AD, mild cognitive impairment (MCI) and healthy control (HC) by imposing structured sparsity on parameter matrix (Jie et al., 2015); ℓ_1,1_-norm regularization term can achieve both structured and flat sparsity (Wang et al., 2011a).

Nevertheless, matrix norms such as ℓ_1_-norm, ℓ_2,1_-norm, and ℓ_1,1_-norm have the natural limitation that they can not take the inherent geometry of the data into account. On this account, we need to select a new distance metric to measure the empirical loss and regularization term. In this paper, we choose the smoothed Wassersetein distance as the distance metric.

### 2.4. Smoothed Wasserstein Distance

Wasserstein distance, originally introduced in Monge (1781), is a powerful geometrical tool for comparing probability distributions. It is derived form the optimal transport theory and is intrinsically the optimal solution of transportation problem in linear programming (Villani, 2008).

In a more formal way, given access to two sets of points $X_{S}={\left\{{\text{x}}_{i}^{S}\in {\mathbb{R}}^{d}\right\}}_{i=1}^{N_{S}}$ and $X_{T}={\left\{{\text{x}}_{i}^{T}\in {\mathbb{R}}^{d}\right\}}_{i=1}^{N_{T}}$, we construct two empirical probability distributions as follows

(7) $${\hat{\mu }}_{S}=\sum_{i=1}^{N_{S}}p_{i}^{S}\delta_{x_{i}^{S}}\text{ and }{\hat{\mu }}_{T}=\sum_{i=1}^{N_{T}}p_{i}^{S}\delta_{x_{i}^{T}},$$

where $p_{i}^{S}$ and $p_{i}^{T}$ are probabilities associated to ${\text{x}}_{i}^{S}$ and ${\text{x}}_{i}^{T}$, respectively, and δ_**x**_ is a Dirac measure that can be interpreted as an indicator function taking value 1 as the position of **x** and 0 elsewhere. For these two distribution, the polytope of transportation plans between $X_{S}$ and $X_{T}$ is defined as follows:

(8) $$U_{{\hat{\mu }}_{S},{\hat{\mu }}_{T}}=\left\{P\in {\mathbb{R}}_{+}^{N_{S}\times N_{T}}\text{s.t.}|\begin{array}{c} Pe=p^{S}\\ P^{T}e=p^{T} \end{array}\right\}.$$

Given a ground metric matrix $\text{C}\in {\mathbb{R}}_{+}^{N_{S}\times N_{T}}$, the optimal transport consists in finding a probabilistic coupling defined as a joint probability measure over $X_{S}\times X_{T}$ with marginals ${\hat{\mu }}_{S}$ and ${\hat{\mu }}_{T}$ that minimize the cost of transport

(9) $${\text{min}}_{P\in U_{{\hat{\mu }}_{S},{\hat{\mu }}_{T}}}\langle C,P\rangle \text{​},$$

where **P** = {*p*(*i, j*), *i* = 1, ⋯, *N*_*S*_, *j* = 1, ⋯, *N*_*T*_} is the flow-network matrix, and *p*(*i, j*) denotes the amount of earth moved from the source $X_{S}$ to the target $X_{T}$. This problem admits a unique solution P^*^ and defines a metric on the space of probability measures (called the Wasserstein distance) as follows:

(10) $$W\left({\hat{\mu }}_{S},{\hat{\mu }}_{T}\right)\overset{\text{def.}}{=}{\text{min}}_{P\in U_{{\hat{\mu }}_{S},{\hat{\mu }}_{T}}}\langle C,P\rangle .$$

Optimizing Wasserstein distance problem requires several costly optimal transport problems. Specialized algorithm can solve it with $O\left(\left(N_{S}+N_{T}\right)\log{\left(N_{S}+N_{T}\right)}^{2}+N_{S}N_{T}\left(N_{S}+N_{T}\right)\log\left(N_{S}+N_{T}\right)\right)$ (Orlin, 1993). To solving the computational problem, recent works have proposed novel method to accelerate the calculation procedure. Furthermore, as a minimum of affine functions, the Wasserstein distance itself is not a smooth function of its arguments. To overcome the above problems, Cuturi (2013) proposed to smooth the optimal transport problem with an entropy term:

(11) $$W_{\gamma }({\hat{\mu }}_{S},{\hat{\mu }}_{T})={\text{min}}_{P\in U_{{\hat{\mu }}_{S},{\hat{\mu }}_{T}}}\langle C,P\rangle -\gamma e(P),$$

where γ > 0 and *e*(·) is the entropy function:

(12) e(P)=−〈P,log(P)〉.

With the entropy term, we can use Sinkhorn-Knopp matrix scaling algorithm to solve the optimal transport problem (Sinkhorn and Knopp, 1967).

## 3. Matrix Regression Based on Joint Wasserstein Distance

In the above formulations, the loss term and estimated variate are characterized via the simple matrix norm. Thus, these models can be easily solved by conventional convex optimization methods [e.g., ADMM (Liu et al., 2010), gradient based methods (Bubeck et al., 2015), and reweighted iterative methods (Nie et al., 2010)]. However, they do not take into account the geometry of the data through the pairwise distances between the distributions' points. Accordingly, these models often achieve the suboptimal results in cognitive score predication.

### 3.1. Joint Wasserstein Matrix Regression

Comparing with matrix norm, Wasserstein distance can circumvent the above limitation. Therefore, in this paper we propose to use Wasserstein distance to jointly characterize loss term and estimated variate **Z**, which is formulated as

(13) $${\text{min}}_{Z}\sum_{i=1}^{l}W_{\gamma }\left(\left(h{\left(A^{T}Z\right)}^{i}\right)h\left(Y^{i}\right)\right),+\lambda \sum_{i=1}^{m}W_{\gamma }\left(h\left(Z^{i}\right),0\right),$$

where *h*(·) and **Y**^*i*^ denote the histogram operator and *i*th row of matrix **Y**, respectively. It should be noted that we use the histogram operator to constrain each variable in model (13).

### 3.2. Optimization Algorithm

Solving problem (13) is extremely challenging since it not only includes the composition of *h*(·) and *W*_γ_(·, ·), but also the computations of Wasserstein distance with regard to different terms. Some existing (Genevay et al., 2016; Rolet et al., 2016) algorithms are only suitable for solving Wasserstein distance loss minimization with matrix norm regularizer. To cope with this challenge, we relax the marginal constraints $U_{{\hat{\mu }}_{S},{\hat{\mu }}_{T}}$ in (11) using a Kullback-Leibler divergence from the matrix to target marginals ${\hat{\mu }}_{S}$ and ${\hat{\mu }}_{T}$ (Frogner et al., 2015; Chizat et al., 2016), i.e., (11) is converted as

(14) $$\begin{array}{l} W_{\gamma }({\hat{\mu }}_{S},{\hat{\mu }}_{T})={\text{min}}_{P\in U_{{\hat{\mu }}_{S},{\hat{\mu }}_{T}}}\gamma KL(P|K)+\mu KL(Pe|{\hat{\mu }}_{S})\\ \;+\mu KL(P^{T}e|{\hat{\mu }}_{T}), \end{array}$$

where **K** = exp(−**C**/*upgamma*).

**Algorithm 1:** Optimization Algorithm of our proposed method.

**Input:** the given ADNI data **A** and related cognitive score matrix **Y** and parameter λ
**Output:** model parameter **Z**
1: **Initialization:** **P**^0^ and ${\hat{\text{P}}}^{0}$
2: **repeat**
3: **for** *t* = 1 to *m* **do**
4: Update each **Z**^*i*^ with proximal coordinate descent
5: **end for**
6: Update ${\text{P}}_{\left(1\right)},\cdots \;,{\text{P}}_{\left(l\right)},{\hat{\text{P}}}_{\left(1\right)},\cdots \;,{\hat{\text{P}}}_{\left(m\right)}$ via Sinkhorn iteration
7: **until** convergence

Let

(15) $$f_{{\hat{\mu }}_{S},{\hat{\mu }}_{T}}(P)=\;\gamma KL(P|K)+\mu KL(Pe|{\hat{\mu }}_{S})+\mu KL(P^{T}e|{\hat{\mu }}_{T}),$$

where parameters γ, μ ≥ 0. Then model (11) ultimately becomes the following form

(16) $$\begin{array}{l} \min\;J(Z;P_{\left(1\right)},\cdots ,P_{\left(l\right)},{\hat{P}}_{\left(1\right)},\cdots ,{\hat{P}}_{\left(m\right)})\\ \;=\sum_{i=1}^{l}f_{{\left(A^{T}Z\right)}^{i},Y^{i}}(P_{\left(i\right)})+\gamma \sum_{i=1}^{m}f_{Z^{i},0}({\hat{P}}_{\left(i\right)})\\ \text{ s.t. }Z^{i}\geq 0,\forall i=1,2,\cdots ,m \end{array}$$

where **P** and $\hat{\text{P}}$ denote the flow-network matrix of $W_{\gamma }\left(\left(h{\left({\text{A}}^{T}\text{Z}\right)}^{i}\right),h\left({\text{Y}}^{i}\right)\right)$ and $W_{\gamma }\left(h\left({\text{Z}}^{i}\right),\text{0}\right)$, respectively, and **Z**^*i*^ ≥ 0 means all the elements in **Z**^*i*^ is greater than or equal to 0.

Due to the relax operation in (14), we can straightly utilize the original data **A**^*T*^**Z**, **Y**, and **Z** in model (16). Thus, we do not need to extract the histogram features of data in the preprocessing stage, which makes it suitable for the prediction task in neuroimaging data.

Strong convexity of model (16) is given by the entropy terms *KL*(**P**|**K**). Thus, we propose to solve (16) by block coordinate descent, alternating the minimization with respect to the parameters $\left\{{\text{P}}_{\left(1\right)},\cdots \;,{\text{P}}_{\left(l\right)},{\hat{\text{P}}}_{\left(1\right)},\cdots \;,{\hat{\text{P}}}_{\left(m\right)}\right\}$ and each **Z**_*i*_, which can be updated independently and therefore in parallel. This is summarized in Algorithm 1. We now detail the two steps of the procedure.

*Updating coefficient matrix* **Z**. Minimizing with respect to one **Z**_*i*_ while keeping all other variables fixed to their current estimate yields the following problem

(17) $$\underset{Z^{i}}{\min}KL\left(P_{\left(i\right)}e|{\left(A^{T}Z\right)}^{i}\right)+\lambda KL\left(P_{\left(i\right)}e|Z^{i}\right).$$

Recalling the definition (2), it is easy to calculate the gradient of objective (17) with regard to each **Z**_*i*_. Thus, we can use accelerated gradient descent (Bubeck et al., 2015) to optimize problem (17).

*Updating parameter set* $\left\{{\text{P}}_{\left(1\right)},\cdots \;,{\text{P}}_{\left(l\right)},{\hat{\text{P}}}_{\left(1\right)},\cdots \;,{\hat{\text{P}}}_{\left(m\right)}\right\}$. For fixed **Z**, the update of each **P**_(*i*)_ and ${\hat{\text{P}}}_{\left(i\right)}$ boils down to an OT problem, which can be solved via Sinkhorn iteration (Cuturi, 2013). These steps are summarized in Algorithm 2, where we list the detailed iteration process for each **P**_(*i*)_. For each ${\hat{\text{P}}}_{\left(i\right)}$, we need to replace (**A**^*T*^**Z**)^*i*^ and **Y**^*i*^ with **Z**^*i*^ and **0**.

### 3.3. Convergence Analysis

Following Sandler and Lindenbaum (2011), we can derive the theorem as follow.

**Theorem 1**. *Algorithm 1 converges to a local minimum*.

*Proof*. Algorithm 1 is the alternative iteration with two iteration stage. In the first stage, we can use gradient descent to solve the convex problem (17). Thus it is obvious that it has a feasible solution. And in the second stage, the problem is a sequence of linear programming processes. As shown in (Sandler and Lindenbaum, 2011), there is a feasible solution for every one of them. To sum up, a feasible solution for (16) exists.

$J\left(\text{Z};{\text{P}}_{\left(1\right)},\cdots \;,{\text{P}}_{\left(l\right)},{\hat{\text{P}}}_{\left(1\right)},\cdots {\hat{\text{P}}}_{\left(m\right)}\right)$ is convex, so applying (17) can derive globally optimal **Z**^*k*^ when given a ${\left\{{\text{P}}_{\left(1\right)},\cdots \;,{\text{P}}_{\left(l\right)},{\hat{\text{P}}}_{\left(1\right)},\cdots {\hat{\text{P}}}_{\left(m\right)}\right\}}^{k-1}$, where *k* denotes the iteration time. Besides, linear programming minimizes the flow-network matrix **P** and $\hat{\text{P}}$. Thus, we can find global optimal **P**^*k*^ and ${\hat{\text{P}}}^{k}$ for a give **Z**^*k*−1^. Furthermore, the accelerated gradient descent used to update **Z** and the Sinkhorn Iteration used to update $\text{P},\hat{\text{P}}$ both have been proven converge.

Since the objective in these two stage is the same, $J\left({\text{Z}}^{k};{\left\{\text{P},\hat{\text{P}}\right\}}^{k-1}\right)\leq J\left({\text{Z}}^{k-1};{\left\{\text{P},\hat{\text{P}}\right\}}^{k-1}\right)$, and $J\left({\text{Z}}^{k};{\left\{\text{P},\hat{\text{P}}\right\}}^{k}\right)\leq J\left({\text{Z}}^{k};{\left\{\text{P},\hat{\text{P}}\right\}}^{k-1}\right)$.

In above, every iteration of Algorithm 1 monotonically decreases $J\left(\text{Z};{\text{P}}_{1},\cdots \;,{\text{P}}_{\left(l\right)},{\hat{\text{P}}}_{1},\cdots {\hat{\text{P}}}_{\left(m\right)}\right)$. This objective is lower bounded, and therefore the algorithm converges.       □

## 4. Experimental Results

In this section, we evaluate the prediction performance of our proposed method by applying it to the Alzheimer's Disease Neuroimaging Initiative (ANDI) database (adni.loni.usc.edu), where a plenty of imaging markers measured over a period of 2 years are examined and associated to cognitive scores that are relevant to AD.

**Algorithm 2:** Sinkhorn Iteration.

**Input:** the given ADNI data **A** and coefficient matrix **Z**
**Output:** {**P**_1_, ⋯, **P**_*l*_}
1: **for** *i* = 1 to *n* **do**
2: **K**_(*i*)_ = exp(−**C**_(*i*)_/γ), where *C*_(*i*)_ is the ground metric between ((**AZ**)^*i*^)*T* and (**Y**^*i*^)*T*
3: **repeat**
4: $\;{\text{u}}_{i}\leftarrow \left({\left({\left(\text{A}\text{Z}\right)}^{i}\right)}^{T}/\text{K}{\text{v}}_{i}\right)$
5: $\;{\text{v}}_{i}\leftarrow \left({\left({\text{Y}}^{i}\right)}^{T}/{\text{K}}^{T}{\text{u}}_{i}\right)$
6: **until** convergence
7: **P**_(*i*)_←(_*p*_(*i*)*jt*_)*n* × *n*_, where the (*j, t*)-th element of **P**_(*i*)_ is *p*_(*i*)*jt*_ = *u*_*i*(*j*)_*k*_(*i*)*jt*_*v*_*i*(*t*)_
8: **end for**

### 4.1. Data Description

The data used in the preparation of our work were obtained from the ADNI cohort. As we know, two widely employed automated MRI analysis techniques were used to process and extract imaging phenotypes form scans of ADNI participants (Shen et al., 2010). One is Voxel-Based Morphometry (VBM) (Ashburner and Friston, 2000), which was performed to define global gray matter (GM) density maps and extract local gray matter density values for 90 target regions. The other one is automated parcellation via FreeSurfer V4 (Fischl et al., 2002), which was conducted to define volumetric total intracranial volume (ICV). All these measures were adjusted for the baseline ICV using the regression weights derived from the healthy control (HC) participants. In this study, there are 805 participants, including 186 AD samples, progressive mild cognitive impairment (pMCI) samples, 167 stable mild cognitive impairment (sMCI) samples and 226 health control (HC) samples. In our work, we adopt FressSurfer markers and VBM markers as imaging phenotypes. Furthermore, the longitudinal scores were downloaded form three independent cognitive assessments including Fluency Test, RAVLT, and TRAILS. The details of these cognitive assessments can be found in the ADNI procedure manuals. The detailed information are shown in Table 1.

**Table 1:** Numbers of participants in the experiments using two different types of imaging markers.

	**#Total**	**#AD**	**#pMCI**	**#sMCI**	**#HC**
FreeSurfer	805	186	167	226	226
VBM	805	186	167	226	226

### 4.2. Performance Comparison on the ADNI Cohort

To evaluate the performance of our model, we compare it with the following related methods: **RR** (multivariate ridge regression), ℓ_2,1_ (robust feature selection based on ℓ_2, 1_-norm), **RSR** (Regularized Self-Representation) (Zhu et al., 2015), and **RLRSS** (Robust Low-Rank Structured Sparse Model) (Xu et al., 2017). These comparing methods are all widely used in statistical learning and brain image analysis.

In the experiments, we use ridge regression for the prediction experiment after selecting the top related imaging markers. We tune the hyper-parameter of all models in the range of {10^−4^, 10^−3^, ⋯, 10^4^} via nested five-fold cross-validation strategy, and report the best result of each method. To measure prediction performance, we compute the root mean square error (RMSE) between the predicted score and the ground truth.

**Figure 1:**
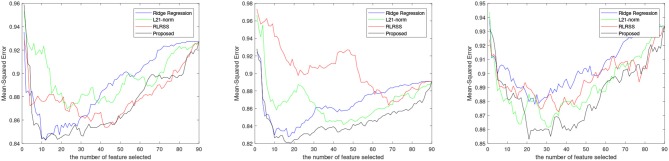
RMSE of four regression methods for VBM memory score prediction.

The average results for each method are reported in Figure 1, while, we also list the RMSE using the top 10 and 30 imaging markers and reported in Tables 2, 3. It can be seen that our ap proach obviously outperforms most of methods significantly. Different matrix norms fit different assumption of the cognitive measures, which makes it enable to uncover part of the correlation of cognitive measures. However, due to the natural limitation of the matrix norms, they fails to uncover the inherent geometry of the cognitive data. As for our proposed method, with the effectiveness of Wasserstein distance, it can well utilize the inherent geometry to reveal the underlying relationship between cognitive measures and neuroimaging markers.

**Table 2:** Prediction performance measured by RMSE with top 10 features.

		**RR**	**ℓ_2, 1_**	**RSR**	**RLRSS**	**Proposed**
VBM	FLUENCY	0.8446	0.9166	0.9044	0.8564	**0.8437**
	RAVLT	0.8376	0.8636	0.8742	0.8943	**0.8263**
	TRAILS	0.9040	0.8823	0.8865	0.8886	**0.8820**
FreeSurfer	FLUENCY	0.8136	0.8387	0.8536	0.8686	**0.8122**
	RAVLT	0.7833	0.8051	0.8337	0.8132	**0.7815**
	TRAILS	0.8416	**0.8181**	0.8433	0.8379	0.8626

The bold values indicate the minimal value in the raw (i.e., the best performance among these methods).

**Table 3:** Prediction performance measured by RMSE with top 30 features.

		**RR**	**ℓ_2, 1_**	**RFS**	**RLRSS**	**Proposed**
VBM	FLUENCY	0.8627	0.8815	0.8879	0.8503	**0.8471**
	RAVLT	0.8543	0.8663	0.8741	0.8736	**0.8327**
	TRAILS	0.8826	0.8618	0.8903	0.8743	**0.8603**
FreeSurfer	FLUENCY	0.8351	0.8323	0.8517	0.8322	**0.8186**
	RAVLT	0.8136	0.7903	0.8154	0.8051	**0.7788**
	TRAILS	0.8295	0.8677	0.8579	0.8335	**0.8274**

The bold values indicate the minimal value in the raw (i.e., the best performance among these methods).

### 4.3. Identification of Informative Markers

**Figure 2:**
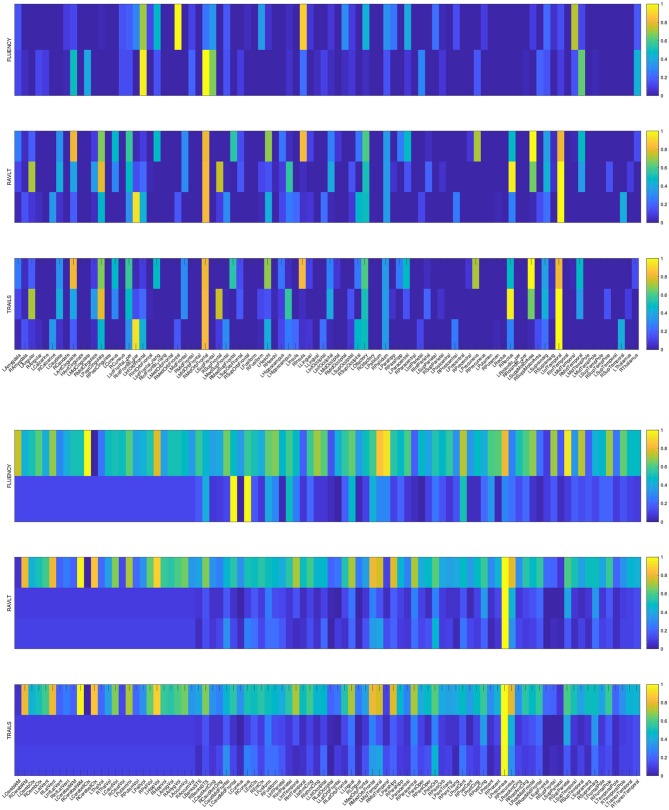
Heat maps of our learned weight matrices on different cognitive assessment scores. The upper panel shows the weight matrices in VBM data and the lower panel shows the weight matrices in FreeSurfer data.

The primary goal of the proposed method is to identify the discriminative AD-specific imaging biomarkers which is crucial for early detection, diagnosis and prediction of AD. Therefore, we examine the neuroimaging markers selected by our method and show it in Figure 2. Visualizing the parameter weights shown in Figure 2 can help us locate the informative markers which play important roles in the corresponding cognitive prediction tasks. As the heat map in Figure 2 shows, different coefficient values are represented in different colors. The yellow polar means a significant effect of corresponding markers on cognitive score performance.

As the Figure 2 shows, the extracted informative imaging biomarks are highly AD-specific and effective for related studies of AD, since it actually meets with the existing research findings. For example, among the top selected features, we found that hippocampal volume (HippVol) and middle temporal gyrus thickness (MidTemporal) are on the top, whose impact on AD have already been proved in the previous papers (Braak and Braak, 1991; West et al., 1994). Furthermore, it also confirms the important significance of the selected neuroimaging cognitive associations to uncover the relationships between MRI measures and cognitive levels.

### 4.4. Visualization of Top Identified Imaging Markers

**Figure 3:**
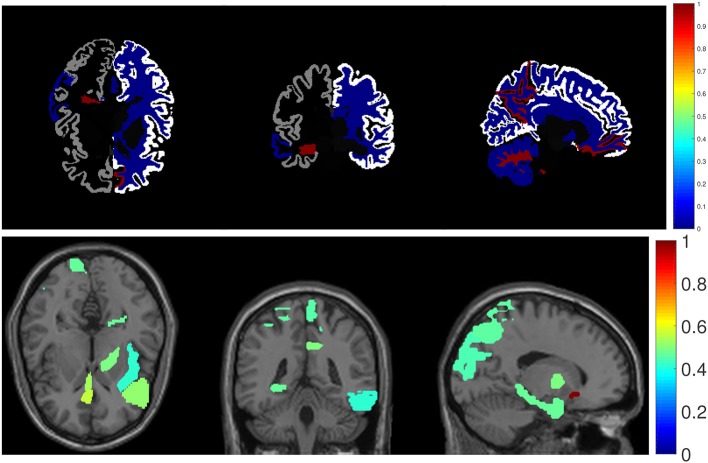
Visualization of top identified imaging markers for RAVLT memory score prediction.

As shown in Figure 3, we also visualize the top ten selected features for RAVLT memory score prediction on brain map as a demonstration. In the brainmap for FreeSurfer, the top 15 brain regions are (in descending order according to the ℓ_2_-norm of feature weights): LPrecuneus, RCerebellWM, LHippVol, RCerebellCtx, RMedOrbFrontal, RLatVent, RCerebWM, RPrecuneus, LParahipp, LMidTemporal, LInfTemporal, RParacentral, LLingual, LPutamVol, RBanksSTS. In the brainmap for VBM, the top 15 brain regions are (in descending order according to the ℓ_2_-norm of feature weights): LRectus, RAntCingulate, LInfFrontal_Triang, RMidCingulate, ROlfactory, RCalcarine, RAmygdala, RRectus, LParahipp, LPallidum, LInsula, RParacentral, LSupOccipital, LInfFrontal_Oper, RMidOrbFrontal.

## 5. Conclusion

To reveal relationship between neuroimaging data and cognitive test scores and predict cognitive score, we proposed a novel efficient matrix regression model which employs joint Wasserstein distances minimization on both loss function and regularization. To eliminate the natural limitation of the matrix norm in regression model, we utilize Wasserstein distance as distance metric. Wasserstein based regularizer can promote parameters that are close, according the OT geometry, which take into account a prior geometric knowledge on the regressor variables. Thus, our proposed method Furthermore, we provide an efficient algorithm to solve the proposed model. Extensive empirical studies on ADNI cohort demonstrate the effectiveness of our method.

## Author Contributions

JY, LL, CD, and HH designed the regression framework and implemented algorithm. JY wrote the manuscript and made the experiment. XW processed the data. JY and XW prepared figures and tables. CD, LL, XY, HH, and LS supervised study and revised the manuscript. All the authors approved the final version of the manuscript.

### Conflict of Interest Statement

The authors declare that the research was conducted in the absence of any commercial or financial relationships that could be construed as a potential conflict of interest.
